# Supplementary material for: Targeted imaging of specialized plant cell walls by improved cryo-CLEM and cryo-electron tomography
Source: Nat Commun. 2025 Dec 12;16:11354. doi: 10.1038/s41467-025-66410-7 (PMC12728224; doi:10.1038/s41467-025-66410-7)
Supplement: Supplementary file 1 — Supplementary Information [file 41467_2025_66410_MOESM1_ESM.pdf]

Targeted imaging of specialized plant cell walls by improved cryo-CLEM and cryo-electron tomography

Daraspe J[1*], Bellani E[2*], De Bellis D[1,2], Genoud C[1,3°], Geldner N[2°]

[1]Electron Microscopy Facility, University of Lausanne, 1015 Lausanne, Switzerland

[2]Department of Plant Molecular Biology, University of Lausanne, 1015 Lausanne, Switzerland

[3]Institute of Bioengineering, Life Science, EPFL, 1015 Lausanne, Switzerland

*These authors contributed equally

°Thes authors jointly supervised the work

Supplementary Information

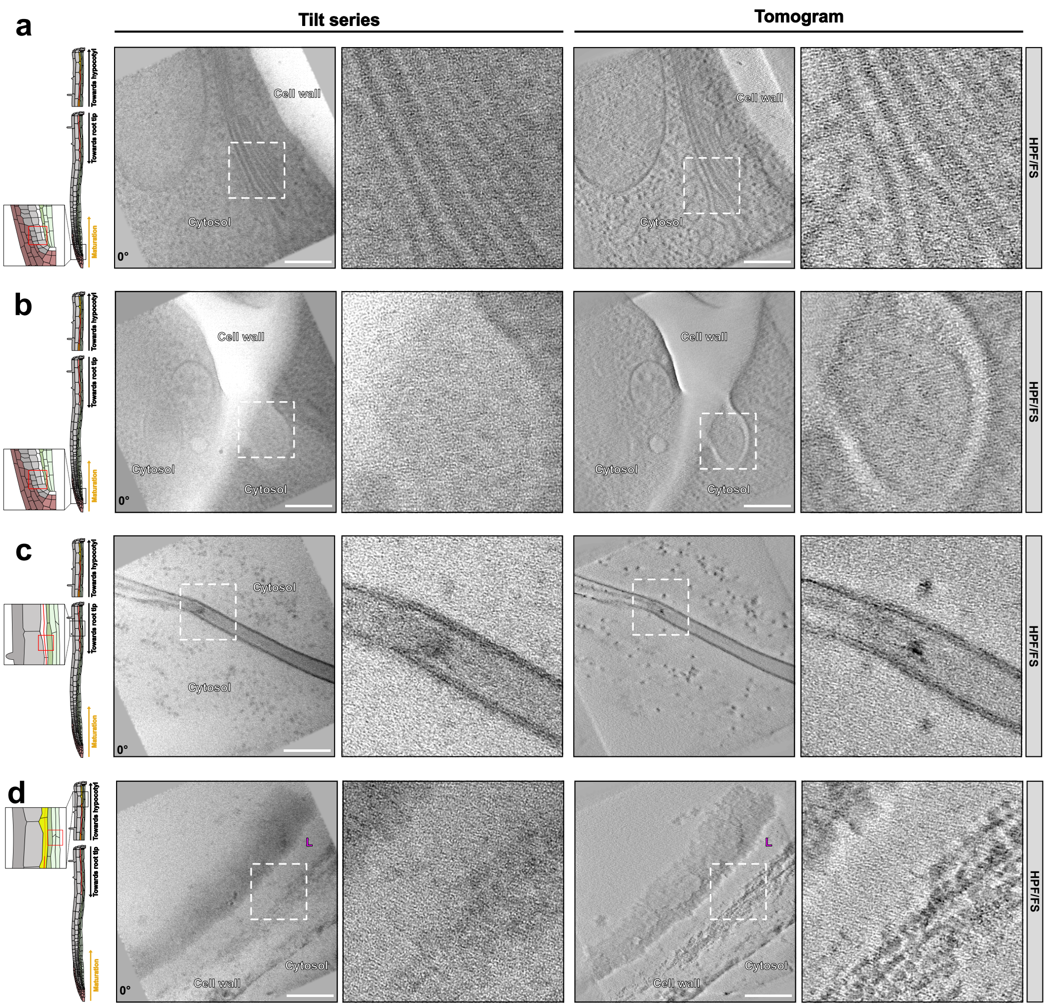

Supplementary Figure 1. **Tomograms of plant structures using HPF/FS**. **a, b** Example of images obtained from the root tip focusing on Golgi apparatus (**a**) and extracellular membrane (**b**) by using HPF/FS. **c** Example of images obtained from the early differentiated root focusing on established CS. **d** Example of images obtained from the late differentiated root focusing on xylem vessels. High-Pressure Freezing (HPF) followed by freeze-substitution (FS).

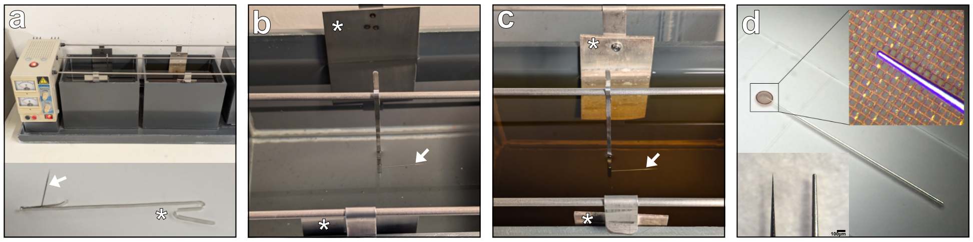

Supplementary Figure 2. **Electroplating setup for silver coating of tungsten EasyLift™ needles. a** Complete electroplating apparatus showing the rectifier on the left, an electrolytic degreasing bath and an electrolytic silver bath with two fine silver anodes (upper panel). Needle holder (asterisk) made with a silver-plated copper strip (3mm width, 0.5mm thickness) maintaining the tungsten needle (arrow). **b** Close-up view of the degreasing bath with the two stainless steel anodes (asterisks) and the needle attached to the needle holder (arrow). **c** Close-up view of the silver bath with the two silver anodes (asterisks) and the needle (arrow). **d** Silver needle tip thickness estimation under a binocular using a 300 square mesh copper grid attached with a transparent tape to a glass slide. Comparison of the original tungsten needle (bottom left) and the silver-plated needle (bottom right) (scalebar 100µm).

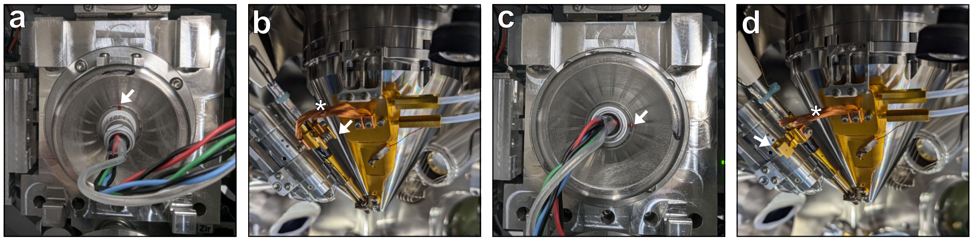

Supplementary Figure 3. **Rotation of the EasyLift™ rod. a** The EasyLift™ rod in the standard position, seen from outside the Aquilos™ 2 chamber. The red mark of the connector position is upward (white arrowhead). **b** The EasyLift™ seen from inside the microscope, showing the clamp (white arrowhead) of the cooling braid (asterisk) placed to the right of the EasyLift™ end. **c** The same view as in (**a**), but rotated 90° CW. The red mark is now on the right, at 90° compared to the previous position. **d** The view from inside the microscope with the 90° CW rotation, showing the downward position of the cooling braid clamp (white arrowhead).

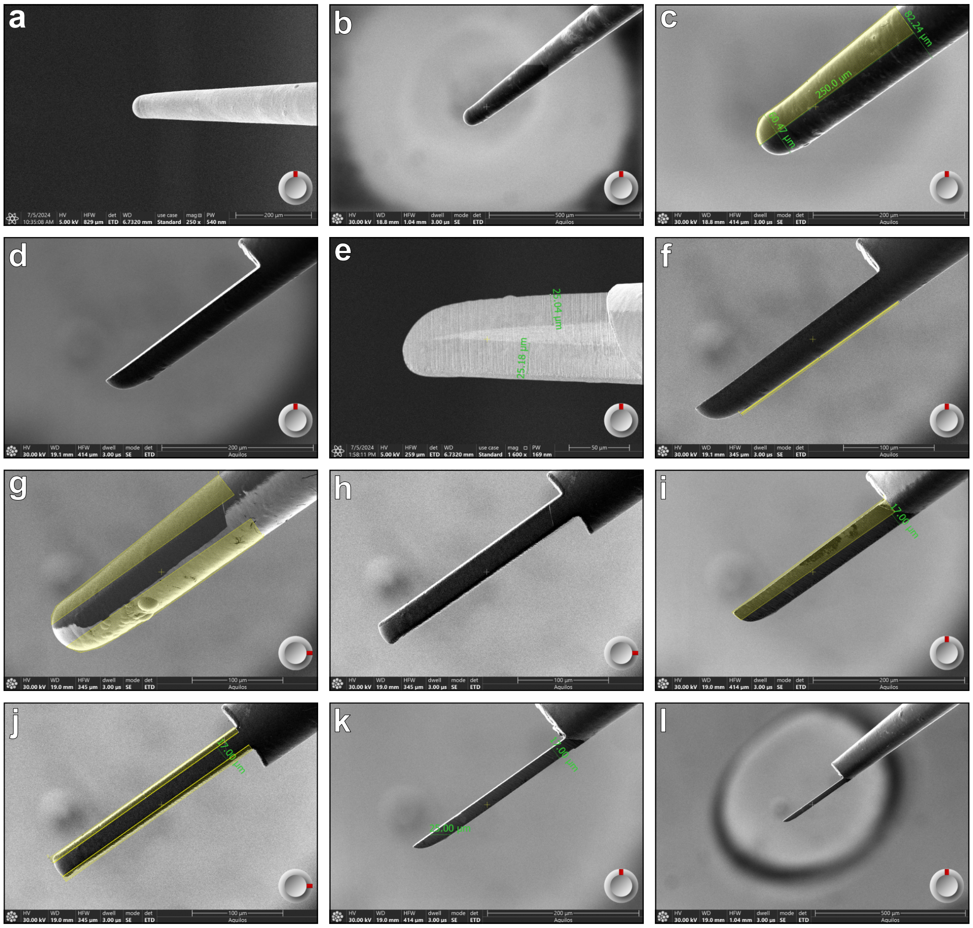

Supplementary Figure 4. **Milling steps for preparing the silver needle. a, b** Initial views of the silver-plated needle in electron beam (EB) and ion beam (IB) views. The bottom right circle with the red mark indicates the EasyLift™ rotation. **c, d** A polygonal pattern is drawn at the needle tip to remove the top half of the needle material. **e** EB view reveals the silver layer thickness around the tungsten core. **f** A thin milling pattern is drawn to create a flat bottom face. **g, h** EasyLift™ rod rotation of 90° CW (red mark to right) exposes the bottom face. Two patterns are drawn to thin down the needle to 25µm. **i** EasyLift™ rod rotation 90° CCW to original position (red mark up). A milling pattern is drawn to remove remaining tungsten and achieve 17 µm thickness. **j** EasyLift™ rod rotation 90° CW enables thinning to 17 µm in the second dimension. **k, l** Final 90° CCW rotation returns needle to the original position. The 17 µm thickness gives a needle width of 25 µm in the horizontal dimension, providing sufficient surface area for robust sample attachment.

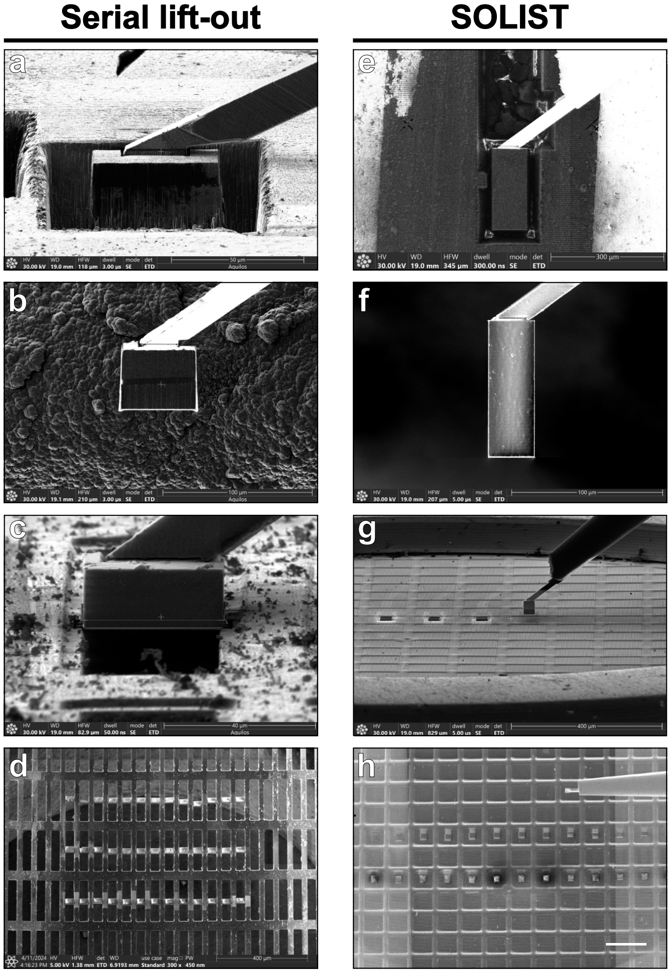

Supplementary Figure 5. **Direct sample attachment using silver needle. a, b, c, d** Serial Lift-Out method. **a** Direct attachment of silver needle to the sample block from a high-pressure carrier using single-pass regular cross-section milling. **b** Lifted-out sample block. **c** Double-sided attachment between grid bar of a 100-400 rectangular mesh copper grid. **d** Full grid view showing lamellae before final polishing. **e, f, g, h** SOLIST method. **e** Direct attachment of silver needle to the sample block from a HPF carrier using single-pass regular cross-section milling. **f** Lifted-out sample block. **g** Sample block lift-out after successful lamella deposition on silver support film. **h** Image of receiver grid showing lamellae before final polishing (scale bar 200 µm).

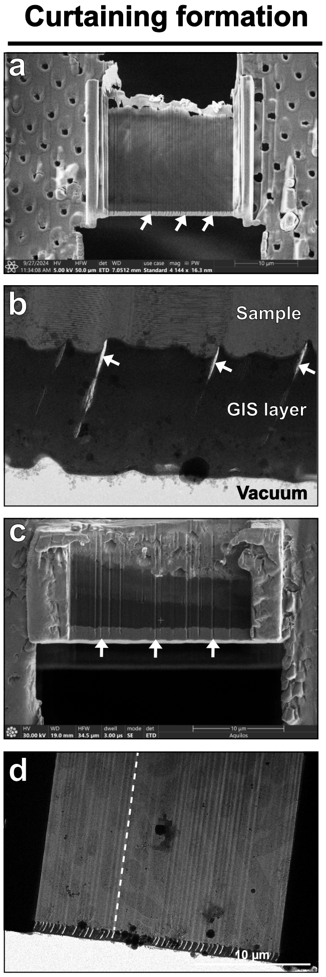

Supplementary Figure 6. **Curtaining formation during lamella preparation. a** Lamella showing a very high curtaining effect after low-current (50 pA) polishing of the milling front without initial thin organometallic platinum deposition on top of the lamella. The organometallic platinum layer shows a high number of cavities (white arrows). **b** TEM view of the same lamella revealing void formation within the organometallic platinum layer (white arrows). These elongated cavities are consistently inclined at approximately 30° to the lamella surface, directly corresponding to the incident angle of the GIS precursor flux. This void distribution suggests shadowing effects during the deposition process. **c** Organometallic platinum deposition performed perpendicular to the lamella front (achieved by rotating the stage to 30°, aligned to the GIS column) after low-current polishing. Despite the modified deposition geometry, similar voids persist in the protective layer

(white arrows), indicating that deposition angle alone is insufficient to eliminate porosity in the organometallic platinum layer and its presence is not related to a shadowing effect. **d** TEM lamella overview showing the very high curtaining effect (parallel to the white dash line) due to the cavities in the organometallic platinum layer.

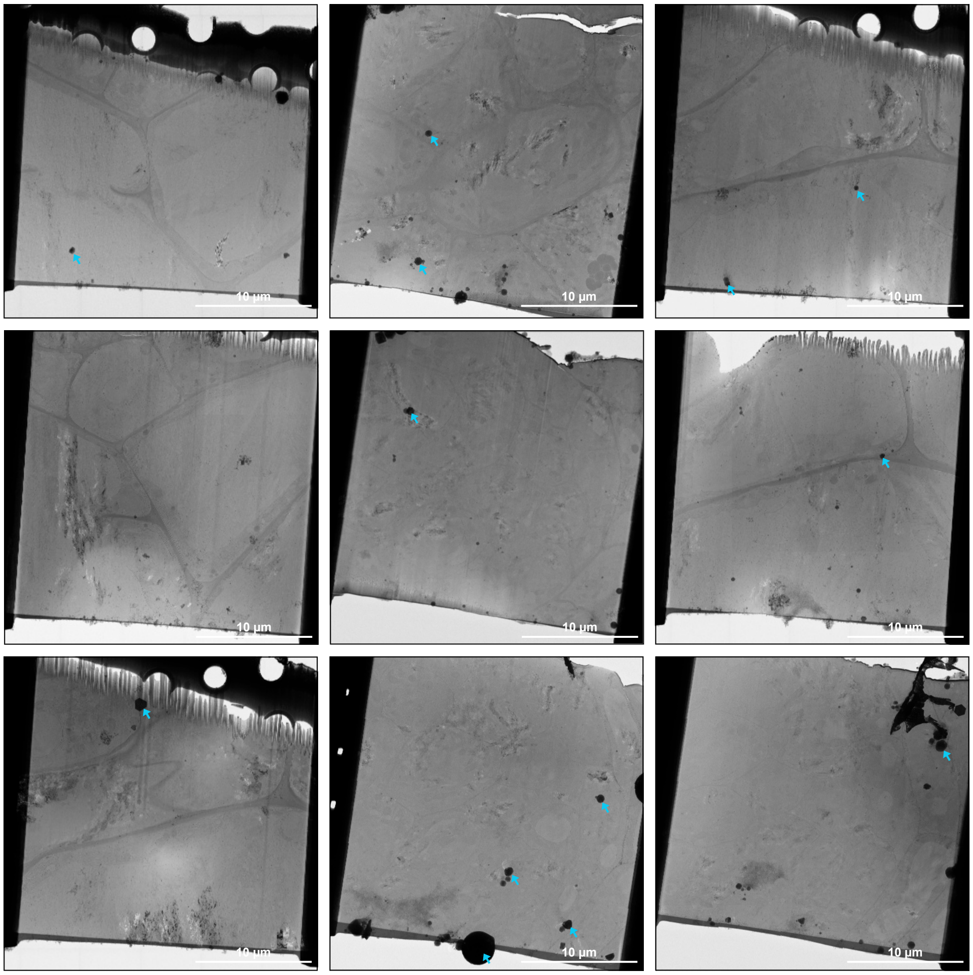

Supplementary Figure 7. **Examples of cryo-lamellae overviews in cryo-TEM.** Lamellae followed the reproducible curtaining reduction strategy described above. Lamellae width is approximately 20 μm, height can vary. Blue arrow – ice contamination on the top of the lamella.
