# Supplementary material for: Targeted imaging of specialized plant cell walls by improved cryo-CLEM and cryo-electron tomography
Source: Nat Commun. 2025 Dec 12;16:11354. doi: 10.1038/s41467-025-66410-7 (PMC12728224; doi:10.1038/s41467-025-66410-7)
Supplement: Supplementary file 2 — Descriptions of Additional Supplementary Files [file 41467_2025_66410_MOESM2_ESM.pdf]

Descriptions of Additional Supplementary Files

Supplementary Movie 1: Tomogram of a Golgi apparatus in root tip showing stacks of cisternae as well as intercisternal structures.

Supplementary Movie 2: Tomogram of a large extracelluar vesicle (EV) with invagination of plasma membrane.

Supplementary Movie 3: Tomogram of the cell wall in cryo-conditions showing the pectin-rich region composed of disorganized fibrils of lower density and electron-dense precipitates. Array of microtubules is visible.

Supplementary Movie 4: Tomogram of established Casparian strip in cryo-fixed sample.

Supplementary Movie 5: Tomogram of vesiculo-tubular structures in a lens shaped space between plasma membrane and the very thin, fibrous wall section between two endodermal cells in a chemically fixed and resin embedded sample.

Supplementary Movie 6: Tomogram of partially formed Casparian strip with few extracellular vesicles in cryofixed sample.

Supplementary Movie 7: Tomogram of fully formed Casparian strip without extracellular vesicle.

Supplementary Movie 8-11: Tomogram of cryo-fixed samples of early suberizing cells showing the organization of the fibrils in cell wall in different orientations.

Supplementary Movie 12-13: Tomograms of Xylem vessels in Cryofixed samples.

Supplementary Movie 14: Image stack of a root segment taken with a cryo-light microscope containing fiducial marks and measurements allowing precise targeting.

Supplementary Movie 15: Movie inside the SEM chamber showing the approach of the needle, the attachment of the block to the needle and the lift-out of the block at double speed.

Supplementary Movie 16: deposition of lamellae on the new grid according to the SOLIST technique. Lamellae of approx. 5 micrometers are cut out of the block and fixed on the grid.

Supplementary Movie 17: attachment strength of the lamellae on the grid according to the modified SOLIST technique tested by pushing the lamella using the silver needle with 10 steps of 200 nm.
